# Elementary Schools’ Response to Student Wellness Needs during the COVID-19 Shutdown: A Qualitative Exploration Using the R = MC^2^ Readiness Heuristic

**DOI:** 10.3390/ijerph19010279

**Published:** 2021-12-27

**Authors:** Hannah G. Calvert, Hannah G. Lane, Michaela McQuilkin, Julianne A. Wenner, Lindsey Turner

**Affiliations:** 1 Center for School and Community Partnerships, College of Education, Boise State University, 1910 University Drive, Boise, ID 83725-1742, USA; michaelamcquilkin@boisestate.edu (M.M.); lindseyturner1@boisestate.edu (L.T.); 2 Department of Population Health Sciences, School of Medicine, Duke University, 705 Broad Street, Durham, NC 27705, USA; hannah.lane@duke.edu; 3 Department of Teaching and Learning, College of Education, Clemson University, 405 Gantt Circle, Clemson, SC 29634, USA; jwenner@clemson.edu

**Keywords:** organizational readiness, education, health, capacity, digital divide

## Abstract

During spring of 2020, the COVID-19 pandemic and accompanying public health advisories forced K-12 schools throughout the United States to suspend in-person instruction. School personnel rapidly transitioned to remote provision of academic instruction and wellness services such as school meals and counseling services. The aim of this study was to investigate how schools responded to the transition to remote supports, including assessment of what readiness characteristics schools leveraged or developed to facilitate those transitions. Semi-structured interviews informed by school wellness implementation literature were conducted in the spring of 2020. Personnel (*n* = 50) from 39 urban and rural elementary schools nationwide participated. The readiness = motivation capacity^2^ (R = MC^2^) heuristic, developed by Scaccia and colleagues, guided coding to determine themes related to schools’ readiness to support student wellness in innovative ways during the pandemic closure. Two distinct code sets emerged, defined according to the R = MC^2^ heuristic (1) Innovations: roles that schools took on during the pandemic response, and (2) Readiness: factors influencing schools’ motivation and capacity to carry out those roles. Schools demonstrated unprecedented capacity and motivation to provide crucial wellness support to students and families early in the COVID-19 pandemic. These efforts can inform future resource allocation and new strategies to implement school wellness practices when schools resume normal operations.

## 1. Introduction

More than 50 million children and youth attended public elementary and secondary schools in the U.S. in 2019 [1]. For many of those students, including the 22 million who receive school lunch at free or reduced price [2], schools are not just settings for educational services, but also for receiving crucial wellness services. According to the Centers for Disease Control and Prevention (CDC), “Aside from a child’s home, no other setting has more influence on a child’s health and wellbeing than their school” [3]. By 25 March 2020, all public K-12 school buildings were closed due to COVID-19 [4], and the instrumental roles that schools played in continuing to meet the basic physical health and safety needs of students, families, and communities garnered national attention.

Schools are crucial settings for promoting student health and wellbeing [5,6,7,8,9]. In 2014, the CDC and the Association for Supervision and Curriculum Development developed the Whole School, Whole Community, Whole Child (WSCC) model to highlight the importance of addressing the multifaceted needs of children and youth to foster optimal learning and development [10]. The model highlights 10 components that facilitate health and academic achievement, including social-emotional support (e.g., counseling and curricula), healthy nutrition (e.g., school meals) and physical activity opportunities (e.g., physical education, recess, classroom activities) [11]. These services foster healthy behaviors and development, as well as academic learning [12,13,14]. Despite the established value of a whole child approach to learning, the ability of schools to prioritize non-academic outcomes is limited [15,16] due to resource constraints and other challenges when implementing WSCC-related policies and programming [17]. These challenges are particularly prominent for schools serving children in higher-poverty areas or belonging to minoritized racial/ethnic groups, who most need school-based services.

The pandemic exacerbated these needs, widening existing disparities in food insecurity, technology access, and parental support for students [18,19,20,21]. The response of school leaders and personnel during the pandemic was not without challenges; however, it also illuminated the strengths of school systems in meeting students’ needs. Understanding the factors which underlie school successes in supporting student wellness during COVID-19 can inform the provision of federal, state, and local resources for schools to implement the WSCC model in the future.

### 1.1. Theoretical Framework

Readiness is recognized as a needed precursor for an organization such as a school to adopt and continue implementing an innovation [22,23,24,25,26]. The R = MC^2^ heuristic, proposed by Scaccia and colleagues [27], suggests that an organization’s readiness (R) to implement an innovative policy, program, or practice results from the organization’s motivation (M) for the change, its general capacity (C), and its innovation-specific capacity (C) [27]. In short, readiness is a product of an organization’s willingness and ability to put an innovation into place. For this study, the pandemic served as a uniform change agent across all schools. The innovation encompasses each school’s unique response to the transition, while operating within the constraints of the federal, state and local context.

Motivation to adopt a new program, practice, or policy relies on the perceived benefits and drawbacks to using said innovation, defined by six factors: relative advantage, compatibility, complexity, trialability, observability, and priority [27]. Although mandated closures meant that most school leaders did not have a choice other than to transition to remote schooling, understanding what motivated school stakeholders’ efforts can inform implementation of wellness services in the future.

General capacity is “the context, culture, current infrastructure, and organizational processes at play where an innovation will be introduced” (p. 4, [27]). General capacity (i.e., culture, climate, organizational innovativeness, resource utilization, leadership, structure, and staff capacity) informs whether an organization universally adapts well to change, and does not depend on resources that facilitate a specific innovation [27]. Urban and rural schools’ general capacity for wellness initiatives is not well-understood. Describing this capacity in the context of a substantial innovation (transitioning to remote provision of services) could be instrumental in informing stronger, more specific recommendations for implementation of wellness initiatives in schools.

Innovation-specific capacity refers to the resources (e.g., financial, tangible) and human capital needed to successfully implement a particular intervention. Activities for building specific capacity are distinct, but can vary in complexity based on the innovation being implemented. During COVID-19, schools’ ability to leverage new and existing resources, knowledge, and relationships were likely critical for providing services, and could yield sustained capacity for in-school supports over time. Factors affecting innovation-specific capacity include innovation-specific knowledge, skills, and abilities; having a program champion; specific implementation climate supports; and interorganizational relationships [27].

### 1.2. Purpose

This qualitative phenomenological study—conducted in the months after schools initially closed due to the COVID-19 pandemic—explores schools’ innovative solutions to provide health and wellness support to students. Through the voices of 50 staff members at 39 elementary schools, we describe how schools adapted services for students and families while prioritizing community safety. We use the R = MC^2^ heuristic to define innovations and describe themes related to schools’ motivation and capacities for implementation.

## 2. Materials and Methods

This study is part of a broader explanatory sequential mixed methods study examining the implementation of wellness practices in elementary schools across the United States. The study had two phases: (1) a nationally representative survey, and (2) follow-up semi-structured interviews with survey participants recruited through stratified sampling and snowball sampling. The current analyses focus on interview questions related to schools’ transition to remote provision of services in response to COVID-19. Interviews were conducted between April and June 2020.

### 2.1. Sampling and Recruitment

Demographic information for the 556 schools in the survey sample was obtained from the National Center for Education Statistics Common Core of Data [1], including locale, which was used to stratify the sample into rural and urban schools for interview recruitment. Urban and rural schools were selected because they are often attended by students living in high-poverty areas or belonging to a racial/ethnic minoritized group. Emails were sent to 153 personnel from rural schools, and 110 from urban schools. Snowball sampling was employed by asking initial participants to provide contact information for additional staff who might like to participate. Ten participants who originally responded for interviews did not complete the scheduling and consent process, for undisclosed reasons. All participants received a 50 USD e-gift card.

### 2.2. Data Collection

Fifty school personnel (hereafter: “participants”) from 39 schools consented to interviews, including 11 referred via snowball. All U.S. regions were represented. School and participant characteristics are listed in Table 1. In comparison to the full sample, the interview sample had fewer schools in the lowest socioeconomic tertile (30.8% versus 41.1%) and more schools in the middle socioeconomic tertile (41% versus 36.6%). The interview sample had a greater proportion of schools serving majority white students (48.7% versus 34.3% in the full sample). Additionally, given the targeted sample of rural schools, a greater proportion of schools in the interview sample had smaller student enrollments.

The interview guide was initially developed to align with the survey and further explore implementation of wellness practices; it was then adapted to explore participants’ perceptions of their role and their school’s role in wellness promotion in the context of COVID-19. The final guide included three topics: (1) schools’ ongoing COVID-19 response; (2) wellness initiatives in the prior school year; (3) future wellness needs and priorities as children return to school. This analysis reflects the first topic (see Supplementary File S1 for interview guide questions).

Given the unprecedented situation and timing of interviews while schools were transitioning to—or continuing and adjusting to—online learning, the guide did not reflect a particular framework; rather, it inquired about participants’ roles in their school’s COVID-19 pandemic response, with the question “What roles has your school played in the community to support student health and safety as a result of the pandemic?” followed by probes for details on school meal distribution, and follow-up questions regarding schools’ motivation, preparedness, leadership involvement, and resources to fulfill these roles.

Interviews were conducted via Zoom or phone call by a single, trained research assistant (MM) after the initial email contact to establish the purpose and time of the interview. After MM reviewed her credentials and the interview purpose, participants were given the opportunity to ask questions about the research and ensure they had a private space to talk prior to the start of the recorded interview. Following the interview, the interviewer documented contextual information and captured initial observations [28,29]. Interviews were audio recorded via Zoom and transcribed verbatim. Interviews ranged from 19 to 91 min (average = 42, SD = 13). Participants did not review the transcripts.

### 2.3. Coding and Analysis

De-identified transcripts were coded and analyzed using Dedoose Version 7.0.23 (SocioCultural Research Consultants, LLC, Los Angeles, CA, USA, 2016). We conducted analysis over two iterative cycles, following best practices for qualitative implementation research and qualitative descriptive design [30,31]. Our first cycle used open coding, applying codes iteratively to identify a compatible, practical coding structure. Transcripts were first divided into excerpts by question. Subsequently, three coders independently reviewed excerpts across a subset of transcripts to develop an axial coding structure, using memos to document coding decisions as well as emerging patterns within and across questions [30]. Coders met repeatedly to discuss these subsets, including areas of overlap between questions and modifications to code definitions. First cycle coding revealed two separate sets of codes: (1) description of innovative roles schools took on during the pandemic response, and (2) factors that influenced schools’ ability to take on and maintain those roles. These findings, as well as our understanding of schools as complex organizations, informed the use of the R = MC^2^ heuristic for our second cycle. We adapted our codebook to include relevant updated constructs described by Wandersman and Scaccia in their 2018 report [32] and iteratively modifying definitions for innovation, motivation, and general and innovation-specific capacity to be school-specific. Once a stable set of code definitions emerged (see Table 2) and coders demonstrated consistency on 20% of transcripts, a single coder applied readiness codes to each excerpt, a second coder double-checked a subset of excerpts, and all three coders met as disagreements emerged. To capture themes within constructs and compare across rural and urban schools, we used extensive memo-ing, memo-linking, and team debriefs. Data saturation was reached and no additional sampling was needed. Participant checking was not used. A detailed audit trail was kept throughout the coding process (available upon request).

## 3. Results

We present our findings in two sections: First, we define the innovative approach of schools to meet students’ academic and wellness needs. Second, we describe components of schools’ readiness for innovative approaches, including themes related to motivation, general capacity, and innovation-specific capacity.

### 3.1. Innovation: A “Network of Support”

**Figure 1:**
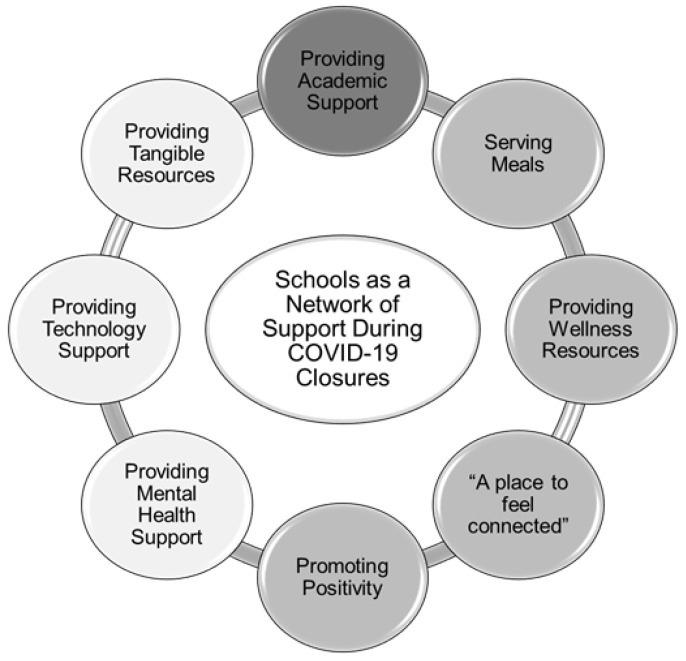
Components of schools’ network of support. Note. Bubbles with darker shading reflect a higher frequency of mentions by participants.

Participants described many important non-academic roles of schools in protecting the health and safety of students during COVID-19, which we conceptualize as a “network of support” (Figure 1). Schools leveraged technology not only to support academics, but also to continue and expand wellness-related services provided during the school year.

In addition to providing support for virtual learning through distribution of laptops, Wi-Fi hotspots, learning packets and use of learning management platforms, the four most-frequent components of schools’ network of support included:

#### 3.1.1. Serving Meals

Meal service occurred in nearly every school district, but varied in scope and approach. Participants described innovative pick-up and/or delivery models for serving nutritious meals to students, especially those with the highest need, including grab ‘n’ go meal pickup or delivery to neighborhoods and homes using school buses.

#### 3.1.2. Providing Wellness Resources

Schools provided information and resources to help keep families safe from COVID-19 as well as staying physically, mentally, and emotionally well. Resources were distributed on the same platforms as virtual learning. A community assistance staff member at an urban school shared, “The school has developed a virtual wellness center. They gathered videos with music, guided reflections, visual relaxations, yoga exercises and mindful videos.”

#### 3.1.3. A Place to Feel Connected

Participants described methods they used to maintain contact with students and families. This mostly occurred via instructional platforms, with follow-up home visits when students could not be reached via web or phone. Participants felt that maintaining this connection was invaluable to students and families. A counselor at a rural school noted, “Reaching out to families made all the difference in the world. A lot of parents said, ‘Thank you for just checking on us.’”.

#### 3.1.4. Promoting Positivity

Participants tried to maintain schools’ role as a place of positivity, consistency, and pride for both students and staff. Examples included birthday car parades, daily motivational social media posts or individual messages, and school decorations:

*We met individually via Zoom with every staff member, [to tell] them what we appreciate about them. I’ve never seen more tears. People are really being reminded of why they do what they do and how gratifying a profession it is. And I got so many comments back like ‘This was so much better than getting a gift card to a restaurant’*

—Urban Principal

Other components of the network of support included providing mental health support through virtual counseling, providing technology support, and providing tangible resources (e.g., gift cards, offering to go grocery shopping).

### 3.2. Readiness

While being “ready” for a crisis of the magnitude of COVID-19 was unlikely, schools described various factors that provided motivation (i.e., commitment or drive to implement innovation) for their network of support, as well as existing or quickly built capacity to carry it out.

#### 3.2.1. Motivation

School stakeholders’ motivation to carry out their version of a network of support fell overwhelmingly into three R = MC^2^ constructs: Simplicity/Compatibility, Priority, and Observability. Codebook definitions and themes for each construct are provided in Table 2. Due to space limitations, not all themes appear in the text, but all appear in Table 2. A sampling of notable quotes for each construct are provided in Supplementary File S2.

##### Simplicity and Compatibility

Many personnel described their schools as being community “hubs” prior to COVID-19, where resources and services (including meals), social-emotional support, and counseling are provided. Thus, schools reported motivation to continue serving as this hub and offering services, adapting delivery models to adhere to their local government’s social distancing guidance.

Schools’ existing partnerships with community organizations (e.g., food banks, mental health providers, churches) made it simpler to step into their new role, as they had previously identified student needs and secured resources to create their network of support. As noted by a physical education teacher at a rural school: “Our community has always been this way. [When] there’s any kind of disaster… we just have these certain organizations in place that…reach out to help the community.”

##### Priority

In terms of serving meals during COVID-19, schools were motivated not only by legal obligations, but a strong moral obligation to offer meals and, in some cases, extend meal programs to non-school aged children, parents, or any individual in need. Regardless of network components, participants acknowledged schools’ imperative to prioritize students’ basic wellness needs, which must be met before they can learn. This was summed up by an urban principal, who stated, “One thing we know is that kids can’t learn if they’re not fed, and not just fed food, but fed emotionally, fed from a security standpoint. That’s why we have tried make things as normal as possible.”

##### Observability

Participants knew that their students’ families would need support, and often described being motivated by the needs of their communities. Many created plans to reach individual students whom they knew would have limited or no access to the network of support (e.g., printing learning packets, providing hotspots for students without Internet access, routing buses to deliver meals to families with transportation barriers).

#### 3.2.2. Capacity (General)

General capacity (attributes of functioning schools) constructs that influenced schools’ abilities to provide the network of support included: process capacities, resource utilization, staff capacities, internal operations, and leadership.

##### Process Capacities

There was little preparedness, planning, or formal evaluation of the network of support throughout COVID-19 school closures, but schools quickly took action without hesitation:

*Schools are general very reticent to change, but [we] really had to adapt quickly…If something didn’t work, we brainstormed that day, and tried something new the next day. We were not prepared at all, [but we] became prepared. When it’s all over, I think we’ll look back and go, ‘wow, we can pat ourselves on the back.’ There’s a lot to be proud of.*

—Urban Principal

Participants described factors that supported or hindered their initial actions. Factors that supported initiation of the network included: having existing infrastructure (e.g., technological systems, staff, programs), team decision making, hands-on leadership, and lessons learned from other schools/districts. Hindering factors included a lack of existing infrastructure and constant decision changes and/or slow decision making by state or local leaders.

##### Resource Utilization

Technology was the most critical resource for the network of support. Helping families overcome anticipated technology barriers was a high priority during the initial weeks of closures, including distributing laptops and/or hotspots to families, setting up parking lot hotspots, and providing technical support to families. Despite these efforts, several participants noted that some students were simply unable to be reached due to limited cell service or low digital literacy. Learning management platforms (e.g., Google Classroom, Class Dojo) were also key resources. Some schools already had these platforms in place, whereas others had to purchase and/or learn to use them. Beyond technology, another resource advantage was existing infrastructure that could be adapted or expanded, rather than started from scratch, such as backpack programs, counseling services, and food and staffing for meal service. As a secretary at a rural school noted, “Because we already had that [backpack] program going, it was just a matter of replenishing those cupboards. Other than that, it’s been pretty smooth.” Participants also described the broad availability of resources from other organizations and districts around the country due to the universality of school closures.

##### Staff Capacities

Many staff members such as teaching assistants, transportation and administration staff, and other non-classroom teachers “stepped up to the plate”, taking on new roles to keep operations going. Staff primarily pivoted to helping with meal preparation and distribution, ensuring that those programs were well-supported. Additional roles included identifying and connecting students to community resources and providing technical support. These new roles kept staff employed, but also enabled more access to students and a better understanding of schools’ non-academic role.

*People always take very seriously the academic part of our mission, but I’m not sure that staff are so focused on how kids are feeling, what they’re going through, what their home life looks like. That gets compartmentalized, so the school nurse, the school counselor, or school psychologist, they worry about those things, and everybody else does their job. In this situation, we’ve gotten a much broader view of our jobs. Our [Spanish and art] teachers have gotten much more involved in finding out what’s happening with kids at home*.

—Urban Principal

##### Internal Operations

While remote methods of communication among staff and between leadership and staff were not ideal, communication was critical, particularly in the early weeks of school closures, as decisions changed quickly and information needed to be shared expediently.

Participants reported the importance of teamwork to develop meal distribution protocols, plan lessons, and identify students who needed additional support. Participants described forming new teams to solve new problems, such as nurses and cafeteria staff developing contact-free meal pick-up protocols, classroom and specials teachers integrating lesson plans, and technology teachers supporting other staff members with virtual platforms.

By necessity, families became essential members of the school’s internal operations, and while schools increased their communication with parents, the expectation of “parents as partners” was not always realistic due to various barriers, such as language barriers, work schedules, lack of internet, frequent mobility and changing phone numbers. Staff members relied on teamwork to reach out to parents, employing both broad (e.g., RoboCalls, surveys) and individualized (e.g., targeted calls or visits to families who had not engaged) strategies. As an urban principal observed, “It’s a big team effort to keep up with kids who aren’t participating. There’s a team that meets every week, we talk about those kids who haven’t been able to participate to any degree. We just want to make sure they’re safe, we want them to participate”.

##### Leadership

Perceptions of leadership varied among participants. Local leaders (e.g., principals, food service directors, superintendents) were perceived positively, while there were mixed opinions about non-local leadership. Problems arose when leaders were less present or involved, constantly changed decisions, or had inadequate communication with staff. A principal at a rural school summarized, “The district did a great job providing what was needed on the ground. I feel like the state and federal guidance and clarification and funding were, um, unclear at best”.

Positive leadership actions overlapped with process capacity and internal operations themes, including swift communication about decisions, involving staff in decision making, and sharing resources as they became available. Leaders also provided emotional support and stability for staff and students. As a principal at an urban school noted, “[I’m] trying to be the liaison between the changing state directives and the district directives and then getting them out to our teachers in a way that doesn’t overwhelm them. I’d say the biggest thing is just being a cheerleader.” Participants frequently described the ways in which leaders went “above and beyond” to meet students’ needs, such as purchasing learning platforms that were appropriate for young children, calling families to notify them of meal distribution route changes, and facilitating district-wide technology trainings for staff.

#### 3.2.3. Capacity (Innovation-Specific)

Innovation-specific capacity—attributes of schools that facilitate adopting an innovation—affected the network of support, with constructs including: knowledge and skills; having a program champion; a supportive climate; and inter-organizational relationships.

##### Knowledge and Skills

In terms of novel skills and knowledge, technical expertise was critical. Participants felt more prepared for the transition to remote services if their schools had access to an information technology department or specialized technology staff. In the absence of this department, schools relied on staff who happened to be tech-savvy. Some larger districts described advantages, as reflected by a principal at an urban school:

*We’re very fortunate…that we have a technology director who also has a staff of technology integrationists, and every elementary building has a media specialist. All of those people have expertise in distance learning, and were able to problem solve 99 percent of the problems that we’ve run into.*

However, the effect of technology knowledge was two-fold; not only did staff in many instances need to learn new strategies on the fly, but many parents struggled to support their younger children with learning technologies. A participant whose school role was as a Learning Director in an urban school noted, “[Many parents] didn’t know how to turn on and off the iPad versus the Chromebook or help their kid get on the camera… or even put in the Wi-Fi”.

##### Program Champion

Participants described specific staff members who exceeded expectations of their traditional role. When a staff member was a champion for families, often they were described as engaging in activities such as personally running errands to get families food, travelling to students’ homes to provide resources and reassurance, or advocating on their behalf to secure internet access. Some also used social media in innovative ways to keep up morale and engagement, instead of or in addition to being a physical presence. Information technology staff, or others who helped with the digital transition, were often mentioned for being invaluable to meeting student/staff needs and dedicating extra time.

##### Supportive Climate

Supportive climate reflected aspects of staff and community attitudes and culture that affected the network of support. The attitudes toward the network of support emphasized the drive of school administrators, staff, and surrounding communities to band together and do anything necessary to support families, much like first responders in a crisis. A physical education teacher at a rural school noted, “It seems like our school just jumps in when there’s someone [that] needs help. They don’t see a barrier. They just go.” Another theme that facilitated the network was “giving grace” to parents who were frustrated and overwhelmed with the new realities of the pandemic. School staff understood the difficulty of the situation and made sure they were showing support and not over-emphasizing academic expectations. Leaders noted that they were primarily concerned with taking care of their staff’s mental wellbeing, as well as making sure families had everything that they needed to function—most commonly including meals and technology support.

##### Inter-Organizational Relationships

Schools mentioned relying on outside assistance to ensure students and families had access to meals in addition to school meals programs. Numerous organizations were noted as integral to mobilizing meal distribution for families, including local food banks, non-profits, faith-based organizations, restaurants, and food service contractors.

A second relationship that emerged was between schools and internet providers; with household internet access becoming critical for schools to reach students, ensuring internet access for everyone became a mission. Some schools struggled with this relationship while other schools had more success brokering deals with the internet providers for families most in need.

### 3.3. Differences between Rural/Urban Schools

Though all schools had similar innovations in terms of the network of support, some differences in implementation strategies between urban and rural schools were apparent. Urban schools had greater ease providing resources to students, while rural schools had to rely on creative strategies to access students living in distant areas. Although rural school staff tended to see their small size as an advantage, this paired with relying on a greater network of partnerships within their community to “get everyone what they needed”. Table 3 shows a contrast of emergent themes that varied across urban and rural schools.

## 4. Discussion

Our study describes the critical roles rural and urban schools played in supporting student wellness, and the infrastructure and processes that supported these roles during the COVID-19 pandemic. It is unrealistic to think that any school could have been fully prepared for the abrupt transition to a completely remote provision of services; nevertheless, schools did not hesitate to take on these roles. We applied a well-established organizational readiness heuristic to understand what helped and hindered implementation of a network of support for students and families during the pandemic.

Our study indicated that the biggest motivators for schools to provide non-academic services during COVID-19 included simplicity and/or compatibility with existing services and observability, or doing what was “morally right”, making sure students were fed and that families and school staff knew that they were cared for. These efforts were recognized and appreciated by families and staff. In another exploration of COVID-19 school meal delivery, food service directors noted a similar potential “silver lining” of the pandemic: that parents’ increased exposure to school meals allowed them the opportunity to see that meals are more nutritious than previously thought [33]. Our findings support prior studies that also demonstrated the strength of alignment of wellness efforts with the school stakeholders’ motivation for taking on new roles [34,35].

In terms of general capacity, our study is not the only one to bring attention to what one rural principal referred to as the “elephant in the room moving forward;” that is, the digital divide that prohibited students with limited or no technology access from benefiting from schools’ network of support during COVID-19, particularly for students in rural areas and for students of color [20,21]. For this issue, schools with more financial resources that could provide tablets or improved Wi-Fi access had a better ability to meet students’ needs. While schools’ creative solutions described by participants in our study may have been able to minimize this disparity in the short term, it is unclear whether these solutions will be sustainable or sufficient to overcome persisting factors such as home access to Wi-Fi, students having minimal supervision or parents having limited technology skills, and exacerbated economic challenges. Even as face-to-face school has resumed for most, the digital divide continues to put some children behind, and while technology infrastructure was clearly beneficial for schools’ efforts to reach students, schools cannot take on the sole responsibility of filling this gap.

A common theme within process capacities and internal operations was that having established channels of communication for both staff and parents gave schools an advantage. The frequent changes in school operations during COVID-19 necessitated constant communication with parents, and parental receptivity to this communication was viewed as an even more critical factor for student wellbeing during the pandemic closure than pre-pandemic. This is consistent with work showing that parent engagement is crucial for many school initiatives, including wellness-related efforts [36]. Another qualitative study of COVID-19 school meal response reported that many parents were frustrated by unclear and inconsistent communication from schools about meal delivery during the pandemic [37]. Future implementation efforts in urban and rural schools should build upon the efforts made in engaging parents necessitated by the COVID-19 crisis.

Within innovation-specific capacity constructs, knowledge and skills, emergent program champions, supportive climate, and external partnerships were all key factors supporting schools’ readiness. While most schools had no shortage of willingness to help students and families, the capacity that schools could use—existing knowledge, resources, and partnerships—for additional support made a big difference in their success. This also echoes the findings of Jowell and colleagues that partnerships with other districts and community organizations helped schools more effectively navigate food service during the pandemic [37]. Innovation-specific capacities built for the remote provision of services, such as tracking systems for flagging kids in need of support, and building parents’ skills for navigating digital communication, could be leveraged to meet the needs of hard-to-reach students in the future. Themes related to both general and innovation-specific capacity underscored a consistent finding in school-based implementation research: the overlapping presence of strong leadership, staff champions, resources, and a supportive climate is essential for successfully implementing school-based wellness initiatives [35,36,38,39,40,41].

When we contrasted emerging themes for rural and urban schools, only a few differences emerged. These primarily related to differing technology barriers and the advantages/disadvantages of being a small or large school. While they had different strengths, urban and rural schools seemed to have similar levels of readiness for responding to the transition to remote schooling. Few studies have qualitatively explored implementation of wellness practices across school locales, but some have shown that rural schools with successful wellness practices rely on larger networks of community resources to provide wellness services [42,43]. When developing implementation supports for schools, it is important to consider the unique challenges and strengths of urban and rural schools.

While school health advocates and researchers have been promoting the CDC’s WSCC approach for years, an important finding of this study was the recognition of its importance by school stakeholders, parents, and community members. Participants often reinforced the notion that “school is much more to kids than just a place to learn”. In particular, the acknowledgement that meeting students’ mental and emotional health needs was a key purpose of the network of support was striking. These findings are supported by a nationwide survey of school employees reporting that WSCC components, including the mental health of students, were a concern among the majority of respondents [44]. As was further brought to light by the events of 2020, trauma-informed practices, including recognizing the role inequities among racial/ethnic minorities and socioeconomically disadvantaged children play in perpetuating health and educational disparities, warrant serious and sustained attention in school communities [45].

The pandemic has clearly changed schools’ approach to meeting students’ health and wellness needs, with an emphasized focus on supporting the whole child. However, it remains unclear whether schools will have the capacity to sustain or expand those roles long-term. For example, flexible distribution of school meals may be advantageous for increasing meal access in the long run; however, revenue shortfalls from these programs in the first months of pandemic-related school closures [46] suggest that this mode of operation is not sustainable without alternating current school meal reimbursement structures. A recent report described the COVID-19-related closures as a key inflection point for public schools, with the rising public recognition, innovative use of technology, and “new allies” in communities and among parents [47]. As noted in recent work, many opportunities exist for leveraging newly created infrastructure to improve implementation of school wellness initiatives long-term [33,37,47,48].

### Limitations

Our study has several limitations. First, the six sub-categories of rural and urban locales were not equally represented, and only two schools from remote rural regions participated; thus, conclusions about the specific constraints of schools in geographically remote areas could not be fully explored. Second, our investigation did not explicitly apply an equity lens to understand how upstream factors (e.g., hiring practices, segregation, racist policies) influenced schools’ readiness during the pandemic, and hinder/enable capacity post pandemic [49,50]. Third, the interview participants held different roles at the school, and we did not examine potential differences in perspectives according to the various positions. Exploring perceived barriers to implementation according to roles might lead to better-tailored recommendations for layering implementation supports within school organizations. These factors should be the focus of future research.

## 5. Conclusions

While our study provides critical insights from school personnel during the early stages of pandemic response, continuing to conduct rigorous mixed-methods implementation research to understand schools’ organizational climate for implementing the WSCC model, as school operations continue amidst COVID-19, is imperative. Despite frequent discussion of readiness as a pre-implementation factor, Wandersman and colleagues describe readiness as dynamic, positing that capacity and motivation “rise and fall over time” [51]. The extent to which COVID-19 school closures have contributed to the “rise” in schools’ readiness to adopt or continue wellness innovations should be empirically investigated. Our application of the R = MC^2^ heuristic as a theoretical lens should be expanded upon in accordance with other research [52], in order to further operationalize organizational readiness for wellness policies and practices in under-resourced schools. Specifically, exploring which readiness constructs are most crucial for improving adoption and implementation of whole child wellness interventions could inform tailored and equitable implementation supports for rural and urban schools (see [46,53]).

Findings highlight the heroic response of schools to the unprecedented disruption and devastation of the COVID-19 pandemic. School leaders and staff were motivated by a moral imperative to support students and their communities. However, institutional capacity was needed to make the rapid pivot needed to provide critical resources, such as food and internet access, to students. Increasing tangible support and resources (e.g., funding) can ensure that the nation’s public education system provides a network of support to students at all times, not only during times of crisis. As schools face an uphill battle to address inequities exacerbated by COVID-19, the current moment is critical for decision makers to advocate for additional resources for continued implementation and sustainability of WSCC-aligned efforts.

## Figures and Tables

**Table 1:** Characteristics of public elementary schools and interview participants.

Variable	Number	%
**School Characteristics (*n* = 39)**		
Student race/ethnicity		
≥50% Asian	1	2.6
≥50% Black	3	7.7
≥50% Hispanic	5	12.8
≥50% White	19	48.7
Other	11	28.2
Socioeconomic status (% of students eligible for free/reduced-priced meals)		
Higher (<33%)	8	20.5
Middle (≥33% to <66%)	16	41.0
Lower (≥66%)	12	30.8
Not reported	3	7.7
School locale		
City: Large	6	15.4
City: Mid-size	4	10.3
City: Small	9	23.1
Rural: Fringe	9	23.1
Rural: Distant	9	23.1
Rural: Remote	2	5.0
School size (number of students enrolled)		
>650	9	23.1
450 to 649	9	23.1
250 to 449	12	30.7
<249	9	23.1
Region		
West	8	20.5
Midwest	10	25.7
South	13	33.3
Northeast	8	20.5
**Interview Participant Characteristics (*n* = 50)**		
Role at School		
Administrator (Principal/Assistant Principal/Head of School)	20	40.0
Physical Education Teacher	9	18.0
Classroom Teacher	2	4.0
Counselor	3	6.0
Nurse	2	4.0
Administrative Assistant/Office Manager	7	14.0
Other	7	14.0
Gender (self-reported)		
Female	40	80.0
Male	10	20.0

**Table 2:** Codebook definitions and themes for schools’ readiness to implement a wellness network of support.

R = MC^2^ Construct and Definition	Theme(s)
Motivation/Momentum	
**Simplicity and Compatibility.** Extent to which network was perceived as an easy role for schools to fill or within the way school usually does things	**Theme 1:** Schools are often the hub of communities/strategic distribution points for resources **Theme 2:** Pre-existing services were not difficult to adapt or maintain for COVID-19 delivery
**Priority.** Importance of network of support compared to academics	**Theme 1:** State mandates required schools to provide meals to students **Theme 2:** School personnel went above and beyond to extend meal services to the whole community out of a desire to meet basic needs
**Observability.** Ability to see or foresee that providing a network of support was what families needed during COVID	**Theme 1:** Student participation rates in existing programs such as free/reduced price meals made the need for a network of support clear **Theme 2:** Personnel from smaller schools described greater ease in identifying which families had the greatest need
**Ability to Pilot.** Degree to which network can be tested or experimented with	Few excerpts emerged; no themes were identified
**General Capacity**	
**Process Capacities.** Ability to plan, implement and evaluate efforts to meet student needs	**Theme 1:** There was little preparedness for the network of support, and there was a lot of trial and error **Theme 2:** Facilitating factors included: existing technological systems; adequate staff, existing programs or preparedness plans; teamwork; learning from other districts; hands-on leadership; knowing students’ needs; having spring break week to prepare **Theme 3:** Barriers included: lack of systems and technology access; constant decision changes/slow decision making by state/local leaders, COVID-19 safety concerns; uncertainty **Theme 4:** Schools used many informal methods to monitor/adjust the network to better meet student needs, including extensive communication with parents **Theme 5:** Schools used many informal methods to monitor/adjust the network to improve operations or logistics and reduce virus spread
**Resource Utilization.** Ability to use existing funds or technological resources to create infrastructure for student wellness	**Theme 1:** Technology was the most critical resource for supporting students during COVID-19; distribution of laptops and/or hotspots was a high priority **Theme 2:** Some technology barriers could not be overcome, and schools instead delivered hardcover textbooks, flash drives or paper packets via bus. **Theme 3:** Having learning management systems (e.g., Google Classroom, Class Dojo) and more tech-trained staff were advantages
**Staff Capacities.** Having enough staff who were able to take on any role to meet student needs	**Theme 1:** Many staff members took on new roles to keep operations going, minimize number of staff in the building, and remain employed **Theme 2:** Staff primarily pivoted to helping with meal service **Theme 3:** Some staff described new roles: calling students who were not attending class; bilingual staff aiding non-English-speaking parents; connecting students to community resources; providing technical support
**Internal Operations.** Effectiveness of communication networks and teamwork among staff	**Theme 1:** School closures necessitated new methods of communication among staff **Theme 2:** Teamwork and resource-sharing were essential and occurred naturally; staff members teamed up in new ways to achieve their goals **Theme 3:** Caregivers served a key new role in operating the network; communication with families was essential, but challenging
**Leadership.** Effectiveness of school and district leaders	**Theme 1:** Local leadership was perceived very positively, views of non-local leadership (state/federal) were mixed **Theme 2:** Positive leadership actions often overlapped with themes related to internal operations and process capacities, including: (1) being attentive and in frequent contact, sharing decision making without creating “decision fatigue” among staff; (2) providing emotional support for staff and students, including “trusting” teachers and keeping expectations realistic **Theme 3:** Leadership were influential in ensuring students had the supplies and resources they needed
**Innovation-Specific Capacity**	
**Knowledge and Skills.** Ability of staff to create network of support for students	**Theme 1:** Staff had base knowledge, but still experienced a learning curve **Theme 2:** As noted in internal operations, parents become key parts of organization who also needed knowledge and skills to facilitate student success; lack of parent knowledge was a barrier
**Program Champion.** Specific people within the school who are particularly promotive of network	**Theme 1:** While teamwork was often noted, sometimes individuals who excelled in filling new/existing roles were mentioned as leaders
**Supportive Climate.** Staff attitudes, parent attitudes, and examples of culture, norms or values that facilitate network	**Theme 1:** Staff were willing to do “whatever it takes” to support families, many spoke that taking care of each other was part of the school culture **Theme 2:** Meeting basic needs was a primary concern of school staff, rather than over-emphasizing academics
**Inter-organizational Relationships.** Support for network from other schools, community partners, volunteers, other external organizations	**Theme 1:** Most schools relied on local food banks, churches, state agencies, internet companies, and other organizations to help meet student needs **Theme 2:** Teachers and administrators worked across districts to collaborate and share resources **Theme 3:** Teachers utilized online networks to adapt their instruction and transition to virtual platforms
**Intra-organizational Relationships.** Relationships between administrators, staff and families to support network	Intra-organizational relationships had extensive overlap with process capacities/internal operations; few unique excerpts emerged; no additional themes were identified

**Note:** Constructs of Relative Advantage and Innovativeness were not assessed due to the pandemic forcing decision making regarding adoption. Additionally, Simplicity and Compatibility constructs were combined into one construct, and all Culture and Climate constructs were combined into one construct. Schools used a variety of informal methods to monitor and adjust their processes, including constant communication with families through broad surveys and individual calls/emails/home visits, and observing bus routes. There were frequent changes, particularly in meal service processes, intended to either better reach students (e.g., expanding bus delivery route or locating new sites near public transportation stops) or improve operations and prevent virus spread (e.g., reducing routes, serving multiple meals/day on fewer days).

**Table 3:** Contrasting themes between rural and urban schools for capacity constructs.

Theme(s)	Representative Quote(s)
**Theme 1:** Being a “small” school or in a small district was often viewed by rural personnel to be advantageous during the COVID-19 response. Being small meant having (1) fewer technology and food resources to distribute; (2) more knowledge of individual student/family situations and needs; and (3) a more tightknit staff and communication network. Rural personnel also described the importance of their role as a “hub” of the community.	“Luckily we’re a smaller school, smaller staff. We all work well together anyways. So I think that was a positive for us.”—Rural Physical Education Teacher “We’re kind of a small, small community. So most people just go straight to the boss and they ask the questions and they get the answers they need.”—Rural Principal “Every student received at least a Chromebook if not an iPad, or both, and um laptops for the older kids. So everyone got something…So because we’re so small, I think it was a little bit easier for us to take this on… we’re mighty because we’re small.”—Rural Principal
**Theme 2:** Both urban and rural schools faced technology-related barriers, but rural personnel described unique barriers (e.g., children lived in more remote areas where the distribution of hotspots was not possible). Rural personnel described innovative mitigation strategies, but noted that for some families, the digital divide could not be overcome and they could not be integrated into the network of support.	“I only have one student that’s getting online with me and my team teachers only have 4 students out of our 27. So we’re copying out lesson plans that we’re making. And they’re being placed at the little grocery store that’s in the nearby town, and parents are asked to go to that grocery store and pick up the lesson plans for their students.”—Rural Classroom Teacher “We do have some resources that we put out on Facebook and the web page for activity ideas and things like that to go along with their lessons, but we’re very rural, and we’re very spread out. So we have a lot of students who don’t have access to internet actually.”—Rural Secretary
**Theme 3:** Rural schools depended on a larger network of community partnerships and support (including faith-based and other community organizations and parent volunteers) to meet the needs of students/families, while urban school participants were more likely to describe how school staff came together to meet the needs of students/families.	“One of my volunteers that attends the local Christian church stepped up, talked with her minister, and we did some of the packing of the bags in the church basement. So this has been a blessing…we have excellent community, and they are such caring people.”—Rural School Nurse

## Data Availability

To request access to de-identified data, please contact the corresponding author.

## Supplementary Materials

The following are available online at https://www.mdpi.com/article/10.3390/ijerph19010279/s1, Supplementary File S1: Interview Questions, Supplementary File S2: Table of Representative Quotes.
