# Supplementary material for: Elementary Schools’ Response to Student Wellness Needs during the COVID-19 Shutdown: A Qualitative Exploration Using the R = MC2 Readiness Heuristic
Source: Int J Environ Res Public Health. 2021 Dec 27;19(1):279. doi: 10.3390/ijerph19010279 (PMC8750629; doi:10.3390/ijerph19010279)
Supplement: Supplementary file 1 [file ijerph-19-00279-s001.zip › Supplementary_File_1.pdf]

Supplementary Materials: Survey Questions

Section 1: Demographic Information

1. What is your current role at the school?
2. How long have you been involved in education (how many years)?
3. What is your gender?

Section 2: Daily Life and Work Activities During COVID-19 Shutdown

1. How are you doing at home?
2. What has your role looked like professionally since you've been home?
3. What roles has your school played in the community to support student health and safety as a result of the pandemic?
4. What motivated your school to take on this role?
5. How prepared was your school or school district for these roles? Did you feel like the needed resources were provided? What do you know about the process to get started?
6. What kind of leadership action was there? What types of barriers needed to be addressed?
7. Who has been involved? How has that changed over time?
8. What resources and information were available to help? Who was providing you with that information?

Section 3: Additional Comments
