# Supplementary material for: Elementary Schools’ Response to Student Wellness Needs during the COVID-19 Shutdown: A Qualitative Exploration Using the R = MC2 Readiness Heuristic
Source: Int J Environ Res Public Health. 2021 Dec 27;19(1):279. doi: 10.3390/ijerph19010279 (PMC8750629; doi:10.3390/ijerph19010279)
Supplement: Supplementary file 1 [file ijerph-19-00279-s001.zip › Supplementary_File_2.pdf]

Supplementary Table S1. Codebook Definitions and Representative Quotes for Schools' Readiness to Implement a Wellness Network of Support

R=MC^2 Construct and Definition	Representative Quote
**Motivation/Momentum**<br>*Features of an innovation that contribute to whether people want to do it and commit to making it happen*	
Simplicity and Compatibility<br>*Extent to which network was perceived as an easy role for schools to fill or within the way school usually does things*	"I think it was just helping the community. Um, she also worked with an outside donor um along with our FBLA (Future Business Leaders of America) sponsor had created prior to the pandemic um a meals program, or I don't know, it was called, like a backpack program where the kids had the ability to take home some things and our high school students that were part of that organization have put together nonperishable packs. And um, so those got to go out in the initially in the beginning um and then the school meals were the next layer." –Rural Principal<br><br>"And so we're a small district, there's 13 schools in the district and so usually when something's going on where there's community that needs to be serviced, my school is one that is a hub, because my school is the only one in this area. So they try to choose certain schools so that the whole community is taken care of, if that makes sense?" –Rural Principal
Priority<br>*Importance of network of support compared to academics*	"You know, the State Board really kind of promoted that when this all started. That was one of the things that they were really concerned about is making sure that those kids, especially like free and reduced lunch kids, had access to those meals still. So that was something that came from the top down." –Rural Principal<br><br>"The food service, I mean, there, there's just a desire to serve the families." –Urban Principal
Observability<br>*Ability to see or foresee that providing a network of support was what families needed during COVID*	"My school is 40 percent free and reduced lunch. So it's one of those things where I know certain students like they don't have access to a computer, they don't have access to the internet." –Urban Physical Education Teacher

	“We based what we were going to do and the role we were going to take off the needs of the community, even going as far to provide not only food, but computers and tech support for the students.” –Urban Principal
**General Capacity**<br>*Organizations Characteristics or conditions applicable for any innovation within an organization*	

Resource Utilization<br>*Ability to use existing funds or technological resources to create infrastructure for student wellness*	“There are some families who have internet only on their phones and you know, you can't really do school work on a phone. So right away there was the technology barrier of devices. Then there was the barrier of no internet service or shoddy internet service. So then, I believe it was [WiFi provider] provided us 1,000 hotspots. Then we also created zones in certain parking lots of our schools, of certain schools where families could literally drive their car to the parking lot of this particular school and get really strong internet service in the parking lot because we put like a booster on top of the school.” –Urban Principal<br><br>“With the poverty level… we ended up just having some students who didn't have the ability to even do any of their work because they couldn't get a Chromebook because they didn't have Wi-Fi.” –Rural Assistant Principal<br><br>“We continue to still do pretty much the same thing we were doing as school counselors. We met with students individually for sessions, we still had our group sessions, we had family therapy sessions, we had parent meetings, we did guidance lessons through Zoom, we met with teachers, we did our 504 meeting still. So our role pretty much was the same. It's just everything was done virtually.” –Rural School Counselor<br><br>“[School meals] is something that we've been doing for a long time. So nothing's really changed…We're actually 100 percent poverty in our district. So, every student gets free breakfast and free lunch….so I think we're, you know, pretty well prepared because we've been doing it throughout the summer program as well” –Urban Assistant Principal<br><br>“I think we were more prepared than we thought, you know, by having learning management systems in place and by having ample staff trained in multiple areas. Again, we, we had that stuff, our food, our kitchen and food services department are self-funded, and they do a really, really good job. And so yeah, they had ample resources in terms of financial and human to make all that happen. And we had enough technology to make it happen... I can't imagine if you were in a place that didn't have all these resources, you know, really how do you do it distance?” –Rural Principal

Process Capacities<br>*Ability to plan, implement and evaluate efforts to meet student needs*	"We learned fast. I mean, it was, two weeks of intense planning and a little bit of chaos, and they refined it very quickly. I've really not seen a project like this one, not just with food, but just with everything. Schools are generally slow to change and very reticent to change, but you really had to adapt quickly. So from the very first day changes were made to the way it was distributed, the way that things were set up so that people didn't have to get in contact with other people, to protect the workers. …So we were not prepared at all and really quickly became prepared… I'm real proud of just the whole system." –Urban Principal<br><br>"It's been interesting. I think it's been kind of a wakeup call, like, we need to get better prepared. We need to have some kind of process in place, you know, for something like this. I don't think we were...they're coming together really good, but it's been a long process and a very confusing one. And now that the school year is almost over, I think we've about got it together." –Rural Physical Education Teacher
Staff Capacities<br>*Having enough staff who can to take on any role to meet student needs*	"Yeah, so I think I think everybody in the system has gotten more aware of their added responsibility. So, you know, people always take very seriously the academic part of our mission. But I'm not sure that every staff member was so focused on how kids are feeling, what they're going through, what their home life looks like. A lot of times that gets compartmentalized, so the school nurse, the school counselor, certainly, or school psychologist, they worry about those kinds of things, and everybody else does their, you know, job. I think in this situation, we've gotten a much broader view of our jobs. So, our teachers, our specialist teachers, like like our Spanish teacher, our art teacher, have gotten much more involved in finding out what's happening with kids at home, checking up on kids." –Urban Principal<br><br>"Really, the entire staff [was involved]. Anyone that can reach out to the kids, their homeroom teacher kind of was a first step of, 'I have heard back from this kid, I haven't heard back from this kid. This mom called me and told me they don't have internet' to then our counselor has reached out, our tech guy was obviously instrumental in getting the hotspots. We had bus drivers helping deliver devices to those that were out in the country. We've had aides help get in touch with people, title 1, special-ed teachers, really honestly it's been an all hands on deck kind of thing." –Rural Physical Education Teacher

	“And our coaches, the district coaches put on for teachers some different Zooms on using Zoom. And you know, like breakout rooms because a lot of them had, that was the first time they'd been on a Zoom. How do use the breakout rooms? When would be appropriate to do that when instructing? And get the most out of Seesaw?” –Urban Lead Specialist

Internal Operations<br>*Effectiveness of communication networks and teamwork among staff*	“I think communication [was difficult] just because so much was changing so quickly, was probably the first barrier of kind of getting everybody on the same page and not being reactive. Just sit with the information and understand it before throwing it out there. And I think we've gotten better with that. At the beginning, I will admit it was difficult.” –Rural Secretary<br><br>“We didn't set up Google classrooms for our elementary kids, we expected them to do packets, we expected parents to be, you know, partners with us. But we kind of understand that their parents might not be even here in the community right now.” –Rural Principal<br><br>“We combined our site council and our ELAC meeting, and we asked those parents on there that joined us for the Zoom like, 'What do you think?' A lot of our Spanish speaking parents were saying, 'You know what, we need some tech help. There's so many other people that were just too embarrassed to show up or you know, to trickle in. So, we're like, 'Okay! So, help with Seesaw?' and they were like, 'No, help with turning it on'. A lot of people didn't know how to turn on and off the iPad versus Chromebook or help their kid get access to the camera get on Wi-Fi. But we made sure…we only have four families now in our school that don't have internet access.” –Urban Learning Director

Leadership<br>*Effectiveness of school and district leaders*	“Our leadership, our administrative leadership in our building has been extremely supportive, extremely flexible. Um, and I think that has made all of this much easier for everyone.” –Urban School Counselor<br><br>“Our admin team is constantly in some sort of meeting with other area administration scouring for ideas. They are in constant contact with us and worrying about how we're doing, if we're overwhelmed or stressed out. They have not put added pressure on us, they want to make sure that we're comfortable in what we're doing so we can do the best we can with kids.” –Rural Physical Education Teacher<br><br>“I feel like the district did a great job providing what was needed just on the ground. I feel like the state and federal guidance and clarification and funding were um, unclear at best.” – Rural Principal

**Innovation-Specific**<br>*Characteristics or conditions necessary to implement specific innovations within an organization*	
Knowledge & Skills<br>*Ability of staff to create network of support for students*	"We had, you know, a lot of parents that just weren't um equipped with the skills to help. If we could have seen this coming we could have prepared parents, you know, teach them technology skills"<br>–Urban Principal<br><br>"I was really overwhelmed emotionally about the pandemic itself, I was also likewise overwhelmed and anxious with what to do and how to do it and learning a lot of new things digitally speaking or online speaking. Like I'm learning this week, I'm teaching myself how to adapt PDF worksheets to Google Forms. So that's what I'm practicing this week to send home to my students online, so that they don't have to rely on getting a paper pencil packet from me." –Rural Teacher
Program Champion<br>*Specific people within the school who are particularly promotive of network*	"Every Wednesday night, um the superintendent does a Facebook Live for the entire school community, um and there have been times where there have been 1500-1700 people viewing the Facebook Live. Um, basically just to give families updates on food service, on Chromebook distribution, on lesson plans, on you know, when we might reopen, just all those things. I mean, he's continuing to even though things have kind of died down a little bit and school is almost over, he's continuing to do that" –Urban Principal<br><br>"And one of the biggest volunteers that helped me was one of our school bus drivers. She actually rode with me every three weeks when we would deliver the three-week buddy pack, or she, I shouldn't say rode with me, she drove my car and I was the one that was in and out dropping them off. I knew that we had a lot of rural areas, but some of them I had to utilize someone that was knowledgeable that drove a bus that knew where these houses were and she did a great job. She has been sticking with me and more than happy to help out." –Rural School Nurse
Supportive Climate<br>*Staff attitudes, parent attitudes, and examples of*	"And some of them [parents] have been grateful and say, said, 'we see that the school is, the school district is trying to do what it's supposed to do to provide the service and teach our students.' And I believe we have a spectrum and it's not only black and white. With this

culture, norms or values that facilitate network	pandemic, we have realized that students and parents can experience stress, anxiety, and sadness, you know, different feelings. But knowing that this school... and all the teachers are there to provide the support that they can for students has shown me that many students, I mean, many teachers truly care for their students. And that's really good to see because that's the reason why we're here." –Urban Community Assistant<br><br>"Um, I would just, I don't know, our community's always been this way. Anytime like, there's any kind of disaster or anything like we just have these certain organizations in place that just, you know, reach out to help the community." –Rural Physical Education Teacher
Inter-org Relationships<br>*Support for network from other schools, community partners, volunteers, other external organizations*	"[Department of Health Services] provided good information, you know, to try to help out with a lot of our kids, the food bank was quite a bit involved, ECCO was involved. Um, a lot of the community recreation centers had information to give out to assist a lot of the parents in that community." –Urban Human Services Staff<br><br>"I know that it doesn't just take the school district to make this happen. So there are tons of community supports, whether it be a business or a hospital or a church that is always working with the school district to support them." –Rural Principal
